# Progress made towards enhancement of rheumatology education and practice in Zambia: review of an ILAR-supported project

**DOI:** 10.1007/s10067-014-2624-0

**Published:** 2014-04-22

**Authors:** James Chipeta, Panganani Njobvu, Paul E. McGill, Richard Bucala

**Affiliations:** 1 Department of Paediatrics and Child Health, University of Zambia School of Medicine, P.O. Box 50110, Lusaka, Zambia; 2 The School of Medicine and University Teaching Hospital Malaria Research Unit (SMUTH-MRU), Department of Paediatrics and Child Health, University Teaching Hospital, D-Block, P/B RW1X, Lusaka, Zambia; 3 Medical Department, Maina Soko Military Hospital, University Teaching Hospital, Lusaka, Zambia; 4 Department of Rheumatology, Stobhill NHS Trust Hospital, Glasgow, Scotland; 5 Department of Medicine/Rheumatology, The Anlyan Center, School of Medicine, Yale University, S525, 300 Cedar Street, New Haven, CT 06520-8031 USA

**Keywords:** EPAREP, ILAR, Rheumatology education, Rheumatology in developing countries

## Abstract

The burden of non-communicable diseases such as musculoskeletal diseases in the developing world is often overshadowed by the more prevalent infectious diseases. Generally, there is gross underestimation of the burden of rheumatologic disease in the backdrop of scanty or indeed non-existent rheumatology services in these countries. Local studies conducted in the last two decades in Zambia have documented the increasing burden of rheumatologic conditions in the country. There are unfortunately negligible rheumatology services in the country both at tertiary or primary health-care facility levels. There is thus an urgent need to build capacity for these services so as to improve the care and management of rheumatic conditions. Here, we review progress made by an International League of Associations for Rheumatology (ILAR)-supported project that has run for the past 2 years (2012–2013) with the objective of enhancing paediatric and adult rheumatology education and practice so as to stimulate positive change in practice and related care services in Zambia. During this short time of the project, substantial progress has been made in the areas of paediatric and adult rheumatology services enhancement at the University Teaching Hospital, Lusaka: streamlining of referrals and follow-ups of rheumatology patients, laying foundations for short- and long-term medical education in rheumatology and raising public awareness of rheumatic diseases. The progress made by this grant underscores the suitability of the ILAR mission statement “think global, act local” demonstrating that even with minimum resources and networking, improvement of rheumatology care in developing countries is attainable.

## Background

The burden of non-communicable diseases such as rheumatic illnesses in the developing world is often overshadowed by the more prevalent infectious diseases in these countries [1–4].

Rheumatology education and practice in many Sub-Saharan African (SSA) countries remain to be largely uncharted, rudimentary and imprecise [1, 5]. The manifestations and impact of prevalent rheumatic diseases (RDs) on the individual and on African societies remain to be incompletely understood. Yet, the theoretical and practical importance of careful and deliberate study of these diseases and of trends in their prevalence and manifestations is well-known.

Firstly, clues to the cause of disease may be identified, e.g. lifestyle or dietary factors. Secondly, explanations for regional, seasonal or other differences in prevalence or manifestation of disease could be elucidated, e.g. heritable or environmental. Finally, appropriate diagnostic and management paradigms could be developed to suit the local situation.

These aspirations cannot be achieved unless the health-care system and health-care professionals on the one hand and the general public on the other are appropriately oriented and have the basic understanding of how RDs manifest and what to do about them.

Zambia, with a population of 13 million people, only has two physicians working (part-time) in rheumatology: one is a paediatrician and clinical immunologist, and the other is a physician and rheumatologist. Both have to divide their time between seeing general paediatric/medical patients and rheumatology patients. There is no training program in rheumatology in the country. Traditionally, patients with rheumatic disorders have been managed by general and orthopaedic surgeons, with little change in long-term prognosis for many patients with severe inflammatory arthropathies.

On the other hand, the general public’s understanding of rheumatic diseases is low and clouded by myths, both about causation and remedy. Unfortunately, some of the myths are perpetrated by the observed lack of efficacy of (the suboptimal or inappropriate) treatment offered to sufferers of RDs who consult conventional medical practitioners.

It was against this background that the EPAREP project was conceived in 2010. EPAREP is short for *Enhancement of Paediatric and Adult Rheumatology Education and Practice*. The project is housed at the University Teaching Hospital in Lusaka and is accredited by the University of Zambia (UNZA) School of Medicine (SOM). The project is currently in its second phase, the first phase having run between 2011 and 2012.

## Project aim and objectives

The vision of the project is to contribute to improvements in the management and care of patients with RDs through enhanced rheumatology education and advocacy in Zambia.

The overall aim of the EPAREP project at the University Teaching Hospital in Zambia is to enhance paediatric and adult rheumatology education and training so as to stimulate positive change in practice and related care services in Zambia. The focussed specific objectives of the EPAREP project are as listed in Table 1.

**Table 1:** Enhancement of Pediatric and Adult Rheumatology Education and Practice (EPAREP) project objectives

1. Set up a long-term monitoring service for determining the prevalence, progression and natural history of rheumatic disorders, the impact of such disorders on the individual and on society, and considering best modes of intervention in the Zambian setting	
2. Advance the cause for rheumatology in Zambia by increasing public awareness and knowledge of both paediatric and adult rheumatic diseases and by engaging health services planners and managers to commit more resources to the care of victims of rheumatic diseases	
3. Develop appropriate core curricula for medical students and postgraduate students and incorporate them into the training programme of the school of medicine	
4. Provide a forum for continuing professional development (CPD) for hospital doctors, general practitioners and other primary care providers in order to improve their ability to assess and manage patients with rheumatic diseases and set up a background for improved patient referral	
5. Establish referral links between the clinics in Lusaka with district hospitals, general practitioners and other primary care providers around the country	

## Progress and achievements

### Initial project phase (EPAREP phase 1: 2011–2012)

**Fig. 1:**
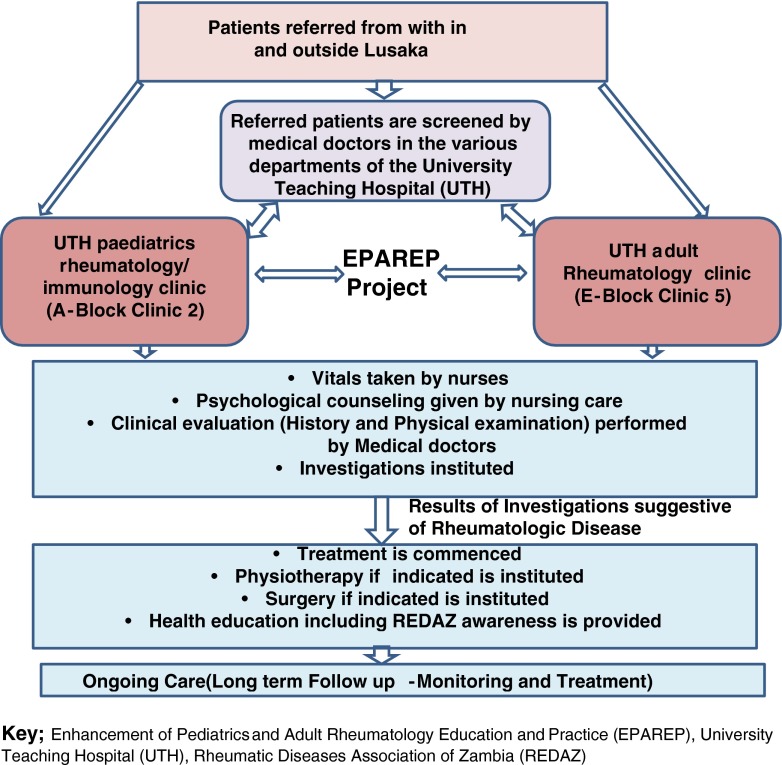
EPAREP established rheumatology patient referral algorithm at University Teaching Hospital (UTH), Lusaka, Zambia. *EPAREP* Enhancement of Paediatrics and Adult Rheumatology Education and Practice, *UTH* University Teaching Hospital, *REDAZ* Rheumatic Diseases Association of Zambia
- Clinical audits and case record reviews of patients seen prior to 2011 were accomplished. This data is enabling comparative analysis of time trends in a relative prevalence of common RDs. Further, four abstracts emerged out of that data were presented at local and international scientific meetings in 2011 and 2012 [6–9].
- In furtherance of the objective of increasing public awareness of both paediatric and adult rheumatic diseases, we established an association for rheumatic diseases—the Rheumatic Diseases Association of Zambia (REDAZ)—which draws its membership from doctors and other health-care professionals with interest in rheumatic diseases, on the one hand, and patients and their relatives, guardians or caregivers, on the other. The association (REDAZ) was formally launched in June 2012 as the first association in Zambia focusing on RDs.
- In addition to spearheading awareness creation and advocacy activities, a core objective of REDAZ is to unite health-care professionals and patients in working together towards the common cause of improving rheumatological services in the country. In this respect, both groups have developed a deep understanding that advocacy for improved service and care can be greatly enriched when each group articulates issues as they affect them and then bring these to the common table for synthesis into awareness and advocacy messages.
- Formation of REDAZ has consequently led to increased understanding and tolerance by patients and their relatives of some of the system inefficiencies, which, for example, sometimes delay appointments or non-availability of service or medicines. Secondly, there has been an increase in the numbers of patients coming to the rheumatic diseases clinics at UTH directly from the community through referral or recommendation by fellow patients or patients’ relatives who are members of the association.
- Regular training and continuous professional development (CPD) activities were also established. This was achieved, firstly, by incorporating rheumatology syllabi into both the under- and postgraduate curriculums of the University of Zambia School of Medicine. In addition, workshops for nurses and physiotherapists, focusing on patient interview, education and counselling, were conducted.

In the first phase of the project, the following were achieved:

- Paediatric and adult rheumatic disease registers were established at the respective clinics at the University Teaching Hospital (UTH), Lusaka. As illustrated in Fig. 1, the patient referral system within the hospital (UTH) was streamlined by creating awareness among hospital doctors about the existence of the two rheumatology clinics in the hospital. This resulted in improved documentation of both paediatric and adult patients; by the end of May 2012, there were 151 patients on the paediatric register and 428 patients on the adult register.

### Current project phase (EPAREP phase 2: 2013–2014)

Most of the phase 1 activities are ongoing and have rolled on into the second phase of the project. The following are the main highlights:

- In phase 2 of the project, there has been further consolidation of the paediatric and adult rheumatology clinics at UTH with continuing increase in patient referral and documented improvement in the numbers of patients attending follow-up reviews (Fig. 1). At the end of July 2013, the adult rheumatology clinic register had 743 patients, while the paediatric register had 230 patients registered.
- More publications have come out of ongoing clinical audits, of which nine were presented at the 2013 Congress of the African League of Associations for Rheumatology (AFLAR) and South African Rheumatism and Arthritis Association (SARAA) in Durban, South Africa [10–19]. In addition, there has been one full publication on juvenile idiopathic arthritis (JIA) [20], and one invited symposium presentation at the 2013 American College of Rheumatology (ACR) annual scientific meeting in San Diego, USA [21].
- Public awareness creation activities have continued through REDAZ. During 2013, REDAZ has exhibited and carried out awareness education at two major events for health-care professionals; the annual scientific conference for the Zambia Medical Association (ZMA) and the National Health Research Conference (NHRC). During the ZMA conference, 168 doctors and medical students were reached, while during the NHRC, 450 health-care professionals (mixed audience) and lay people visited the REDAZ desk and received informational leaflets about the work of REDAZ and EPAREP.
- In addition, in September 2013, REDAZ and EPAREP in conjunction with the Zambia Pediatrics Association (ZPA) ran two radio programs on a community radio station serving the city of Lusaka. The radio station has an immediate audience of about three million residents. The programs focused on providing information on and creating awareness about non-communicable diseases in children with emphasis on JIA, rheumatic fever, sickle cell, trauma and other common childhood rheumatic conditions. These RD awareness campaigns are ongoing and have continued, and by the first quarter of 2014, a further two radio programs have been broadcasting on the main national radio station, the Zambia National Broadcasting Corporation (ZNBC), focusing on common childhood musculoskeletal diseases, highlighting JIA and rheumatic fever.
- Side-by-side with the REDAZ awareness creation activities, EPAREP conducted workshops and symposiums during both the ZMA and NHRC meetings. At the ZMA annual scientific meeting, 60 doctors and 70 students attended a seminar and a workshop on the evaluation and management of RDs and received CPD points or certificates of attendance. During the NHRC, 215 health-care professionals (mixed audience, 50 doctors) attended the symposium on the burden of rheumatic diseases. A further 105 (36 doctors, 20 medical students and 49 other health-care professionals) attended a workshop on the clinical evaluation of paediatric and adult patients with rheumatic symptoms. The 36 doctors received CPD points in addition to certificates of attendance. This brings the total number of doctors trained through EPAREP seminars and workshops to 96, and it is intended to select, for further training and placement as district level referral/processing agents, from among these doctors. The intention is to have district level doctors who are able to diagnose and treat common RDs as well as to identify patients requiring referral for specialist care in a timely manner.
- To consolidate rheumatology education and training for under- and postgraduate students at the UNZA School of Medicine, curricula developed in phase 1 of the project have been followed by the development of teaching materials, which are available in electronic format to lecturers and students.

Table 2 summarizes the current status of the project in relation to set targets and objectives.

**Table 2:** EPAREP project progress status as by end of 2013 with regard to set goals and objectives

Objective	Targets/projected outcomes	Project progress status (by end of 2013)
1. Set up a long-term monitoring service for determining the progression and natural history of rheumatic disorders, the impact of such disorders on the individual and on society, and considering best modes of intervention in the Zambian setting (set up long-term disease activity and impact monitoring service)	a. Disease activity monitoring tools adapted and incorporated into clinic electronic database. b. Four research nurses trained in administration of disease activity monitoring tools. c. Target to have at least five abstract presentations at local or international scientific meetings and two papers submitted or accepted for publication in a peer-reviewed journal by end of current project life	a. This was finalized during the first quarter of 2011 (achievement, 100 %) b. Two trained (achievement, 100 %) c. Nine abstracts [6–18], all of which are published in the July 2013 supplement of clinical rheumatology [19] (achievement, 220 %) and one paper published [20] (achievement, 50 %)
2. Conduct orientation and teaching clinics for hospital doctors, general practitioners and other primary care providers in order to improve their ability to assess and manage patients with rheumatic diseases	a. Target to have at least 20 district level physicians and 40 residents trained through rheumatology focused seminars. b. Target to have 4 nurses and 4 physiotherapists trained. c. Target to have at least 1 nurse, 1 physiotherapist and 2 residents attend some short-term rheumatology elective fellowships abroad	a. 96 trained (achievement, 160 %) b. None trained yet (achievement, 0 %) now planned for 4th quarter) c. Only one doctor attended a 3-week rheumatology elective in SA (achievement, 25 %)
3. Establish referral links between the clinics in Lusaka with hospitals, general practitioners and other primary care providers around the country	a. Have some musculoskeletal disease treatment guidelines developed and in use. b. At least have 50 % of the rheumatology-oriented district level physicians, under the program, implementing district level rheumatology consultations and referrals	a. Developed and part of content of workshops and seminars for doctors (achievement, 100 %) b. Not yet set up formally (achievement, 0 %) and now planned for 2nd and 3rd quarter of 2014
4. Enhance administrative capacity and functionality of the Rheumatic Diseases Association of Zambia and support patient education activities of the patients welfare committee	a. Office assistant to manage REDAZ activities employed. b. Two lay and two nurse counselors trained. c. Four (quarterly) meetings held. d. Conduct at least five TV and/or radio presentations and publish five feature articles on rheumatic diseases in leading Zambian newspapers	a. Engaged (achievement, 100 %) b. Two nurse counsellors trained (achievement, 50 %) c. Four radio presentations made (achievement, 80 %)

The project has been a resounding success by our estimation, with attainment of 100 % or more of set targets for most of the activities by the end of the third quarter of the project. All activities have been ongoing up to the end of December 2013, while implementation of district level rheumatology consultation and referral links is projected to be achieved by the end of 2014. There is, therefore, a high likelihood of achieving targets in most of the remaining activities with the exception of placement of personnel for short-term rheumatology elective fellowships for which the project has no funds or support.

## Discussion

The EPAREP model in Zambia is producing tangible deliverables by fostering enduring partnerships between key stakeholders in rheumatology education and service provision. On the academic front, EPAREP is uniting health-care professionals and training institutions engaged in the training of doctors and allied professionals towards better teaching of rheumatology by helping develop curriculums and training materials. In this respect, EPAREP’s main advantage was the fact that it was conceived within the context of a training program of the Zambian School of Medicine.

In addressing service provision and care of patients, EPAREP is working with the ministry of health and, recently, with the newly established ministry of community development mother and child health (the two government ministries responsible for the provision of health for a majority of the citizens of Zambia) by providing training and CPD for doctors. In this partnership, the ministry of health supports doctors from outside of Lusaka with accommodation and support to attend the EPAREP teaching sessions.

To ensure community participation and advocacy, EPAREP, working with REDAZ, is uniting health-care professionals and their clients in reaching out to the wider Zambian community with essential education awareness messages for the public while, at the same time, lobbying government and health services managers to become more aware of the burden of rheumatic diseases and the need to invest more into their management.

## Conclusion

### Advantages accrued from the ILAR grant

It is an undeniable fact that most grant and funding organisations hardly fund projects addressing non-communicable diseases in developing countries. In this respect, ILAR support has played a pivotal role in the success of the EPAREP project. Availability of the grant, coupled with linkages and collaboration with colleagues from developed countries, enabled the project to widen the scope of what can be done in a resource-limited setting such as ours.

It is our sincere hope that ILAR will sustain these awards to developing countries so as to help bridge the gap of rheumatology services between developed and developing countries.
